# Of the milk sugars, galactose, but not prebiotic galacto-oligosaccharide, improves insulin sensitivity in male Sprague-Dawley rats

**DOI:** 10.1371/journal.pone.0172260

**Published:** 2017-02-16

**Authors:** Priska Stahel, Julie J. Kim, Changting Xiao, John P. Cant

**Affiliations:** 1 Department of Animal Biosciences, University of Guelph, Guelph, Canada; 2 Departments of Medicine and Physiology, University of Toronto, Toronto, Canada; University of Melbourne, AUSTRALIA

## Abstract

**Background:**

Consumption of dairy products reduces risk of type 2 diabetes. Milk proteins and fats exhibit anti-diabetic properties but milk sugars have been studied little in this context. Galactose from milk lactose is readily converted to glycogen in the liver but its effects on insulin sensitivity have not been assessed. Prebiotic oligosaccharides from milk alter gut microbiota and can thereby influence host metabolism. Our objective was to assess the effect on insulin sensitivity of dietary galactose compared to glucose and fructose, and fermentable galacto-oligosaccharides compared to non-fermentable methylcellulose.

**Methods:**

Diets containing 15% of dry matter from glucose, fructose, galactose, galacto-oligosaccharides, or methylcellulose were fed to 36 rats per diet for 9 weeks. Hyperinsulinemic-euglycemic clamps with [3-^3^H]glucose infusion and a steady-state 2-[1-^14^C]deoxyglucose bolus injection were used to assess insulin sensitivity and glucose uptake indices. Tissue was collected in fed, fasted and fasted, insulin-stimulated states.

**Results:**

Galactose increased glucose infusion rate during the clamp by 53% and decreased endogenous glucose production by 57% compared to glucose and fructose. Fed-state hepatic glycogen content was greater with galactose compared to glucose and fructose, consistent with a potentiation of the insulin effect on glycogen synthase by dephosphorylation. Galactose decreased the fecal Firmicutes:Bacteroidetes ratio while galacto-oligosaccharides increased abundance of fecal *Bifidobacterium* spp. 481-fold compared to methylcellulose, and also increased abundance of *Lactobacillus* spp. and Bacteroidetes. Galacto-oligosaccharides did not affect glucose infusion rate or endogenous glucose production during basal or clamp periods compared to methylcellulose.

**Conclusions:**

Galactose at 15% of daily intake improved hepatic insulin sensitivity in rats compared to glucose and fructose. Galactose caused an increase in fed-state hepatic glycogen content and a favourable shift in gut microbial populations. Intake of galacto-oligosaccharides improved the gut microbial profile but did not improve insulin sensitivity.

## Introduction

Epidemiological studies assessing dairy product consumption by questionnaire [1,2] and biomarker analyses [3] have linked increased dairy consumption with decreased markers of metabolic syndrome. Similarly, type 2 diabetes risk is decreased by 9% and 4% in men [4] and women [5] respectively, with each additional serving of low-fat dairy products per day. Some studies demonstrate accelerated weight and fat loss in obese and overweight individuals consuming dairy products [6,7] while others show no effect [8].

Many components of milk may contribute to its anti-diabetic effect. Whey protein in the diet improves glucose tolerance which has been attributed to the insulinotropic effects of increased GLP-1 secretion [9] and amino acid absorption [10]. Dairy calcium improves glucose tolerance to a greater extent than elemental calcium [11] while dietary trans-palmitoleate, a monounsaturated fatty acid found exclusively in ruminant products, is negatively correlated with insulin resistance and dyslipidemia [3]. The milk sugars, lactose and galacto-oligosaccharides, have been studied little in the context of insulin sensitivity, although there is reason to expect positive effects. Lactose contains the simple sugar galactose which, unlike the glucose and fructose moieties of sucrose, passes completely into hepatic glycogen upon absorption [12]. This glycogenic effect may impact whole-body glucose utilization and insulin sensitivity, although such effects have not yet been studied, to our knowledge. In contrast, chronically high intakes of glucose and fructose have long been linked to disorders in insulin sensitivity, partially explained by pancreatic β-cell failure [13] and high lipogenicity [14], respectively. The oligosaccharides of milk, primarily consisting of glucose, galactose, *N*- acetylglucosamine, and fucose monosaccharides [15], are indigestible in the small intestine but are fermented in the large intestine and encourage growth of beneficial colonic *Bifidobacterium* and *Lactobacillus* spp. [16] These favourable shifts in gut microbiota may improve hepatic metabolism and insulin sensitivity in the host by altering colonic short-chain fatty acid production, gut peptide secretion and gut barrier function [17].

The objective of our study was to evaluate effects on insulin sensitivity of the milk sugars galactose and galacto-oligosaccharides. Galactose was compared to glucose and fructose, and indigestible but fermentable galacto-oligosaccharides were compared to indigestible and non-fermentable methylcellulose, all at 15% of otherwise identical diets. We hypothesized that galactose would result in greater insulin sensitivity than glucose and fructose due to its lower lipogenicity and propensity to be stored as glycogen. Galacto-oligosaccharide intake was predicted to improve gut microbial profile by increasing *Bifidobacterium* spp. and decreasing Firmicutes, leading to improvements in hepatic insulin sensitivity.

## Materials and methods

### Rat feeding and housing

Five diets (Research Diets, Inc., New Brunswick, NJ, USA) were formulated to contain, on a dry basis, 22% casein, 23% corn starch, 11% maltodextrin, 2.7% soybean oil, 14.5% lard, 5.5% cellulose, 6.2% vitamins and minerals, and 15% glucose (GLC), fructose (FRC), galactose (GAL), galacto-oligosaccharides (GOS; Cremar, Seoul, South Korea) or methylcellulose (MC). 180 male, Sprague-Dawley rats were acquired from Charles River Laboratories (St.Constant, QC, Canada) at 225 to 250 g body weight in blocks of 24 rats. They were housed in groups of three in a climate-controlled room at approximately 22°C and 80% humidity with a 12-hour light/dark cycle and *ad libitum* access to water. Each group was fed one of the five diets for nine weeks. Weekly body weights were recorded. In the ninth week, one rat per cage was used for the hyperinsulinemic-euglycemic clamp procedure followed by tissue collection, while the remaining two were used for fasted, fed or insulin-stimulated tissue collection. All animal procedures were approved by the Animal Care Committee of the University of Guelph (Animal Utilization Protocol #2446).

### Hyperinsulinemic-euglycemic clamp

Catheters were inserted into the right jugular vein and left carotid artery under isoflurane anesthesia. Rats were given 3 days to recover from the catheterization surgery before undergoing the hyperinsulinemic-euglycemic clamp. Those that lost more than 5% of body weight after surgery were not used in the clamp. Clamps were performed on overnight-fasted, conscious rats. The basal period (-90 to 0 minutes) involved a primed, jugular infusion of 0.4 μCi kg-1 min-1 [3-^3^H]glucose (Perkin-Elmer, Waltham, MA, USA) to quantify basal endogenous glucose production (EGP). Blood was drawn from the carotid artery to measure glucose concentration every 10 minutes during the last 30 to 40 minutes (OneTouch UltraBlue 2, LifeScan Canada, Burnaby, BC, Canada). Approximately 100 μl blood was collected into heparin-containing tubes for future quantification of tracer and insulin.

The clamp period (0 to ~120 minutes) began with an insulin bolus of 20 mU kg-1 min-1 for 2 minutes (porcine insulin, Sigma-Aldrich Canada Co., Oakville, ON, Canada) followed by a continuous jugular infusion of 4 mU kg-1 min-1 insulin and 0.8 μCi kg-1 min-1 of [3-^3^H]glucose. A 25% D-glucose solution was infused at variable rates, adjusted every 10 minutes, to clamp blood glucose at basal levels. Once blood glucose and glucose infusion rates (GIR) were stable for 30 minutes, a 40-μCi bolus of 2-[1-^14^C]deoxyglucose (2-DOG; Perkin Elmer) was infused through the venous catheter. Subsequent blood samples of 80 μl were taken at 7, 10, 15, 20 and 30 minutes in heparin-containing tubes. This procedure for quantification of *in vivo* glucose uptake is modified from White et al., (2016) [18].

Difference in hematocrit between basal and clamp periods was used to disprove anemia due to excessive blood sampling, with less than 10% difference considered acceptable. Plasma was stored at -20°C until further analysis. Rats were anaesthetized by i.v. pentobarbital prior to *in situ* tissue collection of gastrocnemius and soleus muscle, omental adipose tissue, diaphragm, interscapular brown adipose tissue (iBAT), brain and heart. Tissue samples were snap-frozen in liquid N2 and stored at -80°C until further analysis.

### Tissue glucose uptake

Tissue samples were homogenized in 0.5% perchloric acid and centrifuged at 2000 g for 20 minutes. Neutralized supernatant radioactivity was measured for total activity in 2-DOG and 2-DOG phosphate. Radioactivity in 2-DOG was measured after deproteinization by barium hydroxide and zinc sulfate (Sigma-Aldrich Canada). Tissue [2-^14^C]DOG phosphate was calculated as the difference between total and 2-DOG activity.

Glucose uptake index (Rg) was calculated as:

$$\frac{[2-14\text{C}]\text{DOG phosphate tissue}}{\text{AUC}\left[2-14\text{C}\right]\text{DOGplasma}}\cdot \text{glucose plasma}$$

where glucose plasma is the average concentration of plasma glucose after 2-DOG injection, and area under the curve (AUC [2-^14^C]DOGplasma) was estimated from fits of the dual-exponential equation ${\text{A}}_{1}{\text{e}}^{-{\text{k}}_{1}\text{t}}+{\text{A}}_{2}{\text{e}}^{-{\text{k}}_{2}\text{t}}$ to measured [2-^14^C]DOG counts at time points t up to the last sample at tfinal, using the Solver function of Microsoft Office Excel^®^ 2007 [19]:

AUC[2−14C]DOGplasma=A1k1(1−ek1tfinal)+A2k2(1−ek2tfinal)

To account for different concentrations of brown adipose tissue in the interscapular sample, the Rg for iBAT was adjusted for uncoupling protein 1 (UCP1) content detected by western blot.

### Plasma analysis

Heparinized plasma from basal and clamp periods was dried at 65°C after deproteinization by barium hydroxide and zinc sulfate (Sigma-Aldrich Canada). The tracers [3-^3^H]glucose and [2-^14^C]DOG were counted in dried samples using Ultima Gold scintillation fluid cocktail (Sigma-Aldrich Canada) and a Beckman LS6000 liquid scintillation counter (Beckman, Brea, CA, USA). Samples of infusates were also counted. Plasma insulin was quantified by enzyme-linked immunosorbent assay (Crystal Chem, Downers Grove, IL, USA).

### Glucose flux calculation

Glucose fluxes (mmol min^-1^) during the basal and clamp periods were calculated as tracer infusion rate (dpm min^-1^) ÷ specific activity of plasma glucose (dpm mmol^-1^) and were adjusted for body weight to yield glucose fluxes in mg kg^-1^ min^-1^. EGP was calculated as glucose flux—GIR. Percent suppression of EGP by insulin was calculated from the difference in EGP between clamp and basal periods. A portion of the GIR during the clamp compensates for EGP suppression and the remainder compensates for stimulation of peripheral glucose utilization (PGU). Thus, percent stimulation of PGU by insulin was calculated from the difference between clamp glucose flux and basal glucose flux.

### Fasted- and fed-state tissue collection and analysis

*In situ* tissue collection under pentobarbital anesthesia was performed in 8 rats per diet, 3 hours post feeding. Proximal colon (2 centimetres of colon tissue distal to the ileocecal junction) and liver tissue were snap-frozen in liquid N2 and stored at -80°C until further analysis. Liver samples were also collected under pentobarbital anesthesia from 16, 12-h fasted rats per diet, eight minutes after i.p. injection of either 10 U kg^-1^ porcine insulin or the equivalent volume kg^-1^ of vehicle (saline).

Bodies of vehicle and insulin-stimulated rats were frozen before being ground and dried at 65°C for 72 hours to determine moisture, crude protein and lipid (ether extract) content following AOAC procedures [20].

Hepatic glycogen content from fed, fasted vehicle and clamp rats was determined following methods of Liu et al. [21], as the difference in glucose measurement by colorimetric assay (Sigma-Aldrich Canada) with and without 2-hour incubation at 37°C with amyloglucosidase (EC 3.2.1.3.).

### Gene expression analysis

Proximal colon RNA, isolated via the TRIzol method, was reverse transcribed (High Capacity cDNA Reverse Transcription Kit; Applied BioSystems, Waltham, MA, USA). Differences in gut microbial populations were quantified from fecal DNA isolated (MP Biomedicals FastDNA SPIN kit for feces) from fecal samples collected during the 8th week from bedded cages. Primers (S1 Table) were used in RT-qPCR (PerfeCta SYBR Green FastMix; Quanta BioScience, Gaithersburg, MD, USA) with an Applied Biosystems 7300 Real Time PCR instrument. Gene expression was analyzed by the 2-ΔΔCt method [22] with β-actin (liver) and universal bacterial gene (fecal) as the reference genes and presented as fold-change relative to the MC diet.

### Western blotting

Protein concentration of homogenized liver was quantified by BCA assay (Thermo Scientific, Waltham, MA, USA). 20 μg protein was loaded per well on 6 to 10% SDS-PAGE gels, separated and transferred to nitrocellulose membranes. All primary antibodies were from Cell Signalling Technology (Danvers, MA, USA) and incubated at room temperature at 1:1000 unless otherwise stated; total glycogen synthase (GS; EC 2.4.1.11), phosphorylated GS (pGS, Ser641), glycogen phosphorylase (GP; EC 2.7.7.9), and β-actin (1:40,000). Insulin’s ability to suppress inhibitory phosphorylation of GS was calculated as the ratio of phosphorylation states of GS in vehicle:insulin injections for rats in the same cage.

UCP1 abundance in iBAT was detected from 20 μg protein to confirm the proportion of iBAT tissue in the sample. Membranes were then incubated with secondary anti-rabbit (1:10,000) or anti-mouse (1:2000). Bands were visualized with enhanced chemiluminescence (Bio-Rad, Hercules, CA), and imaged using the chemidoc MP imager (Bio-Rad, Hercules, CA). Imagelab software version 5.1 was used to determine background-subtracted band density.

### Statistical analysis

ANOVA was conducted using PROC GLM of SAS (version 9.2; SAS Institute Inc., Cary, NC) according to a randomized block design. Normality was assessed using PROC UNIVARIATE of SAS. If not normal, data were natural log-transformed to obtain *P*-values and treatment differences. Multiple comparisons of all 5 treatments against each other with Tukey adjustment revealed that GLC and FRC were rarely different from each other so, to increase power of the test, GAL effects were compared against the combined effects of GLC and FRC (*P*_GAL v GF_) by orthogonal contrasts. In addition, no meaningful differences were found between GOS and GAL. Due to the presumed prebiotic effects of GOS, it was compared to MC which serves as a negative control due to its non-fermentable nature. The comparison of GOS vs MC performed by orthogonal contrasts is represented by *P*_GOS v MC_. Significance was declared at *P* ≤ 0.05, while *P*-values between 0.05 and 0.1 were considered trends. Data are presented as means ± SE. Means of log-transformed data were exponent-transformed to present in figures and tables.

## Results

### Body composition

After nine weeks of feeding, body weight was greater with FRC consumption compared to GLC (GLC: 497 ± 11.0 g, FRC: 551 ± 15.6, GAL: 502 ± 10.5), which was due to a higher fat mass (GLC: 52.7 ± 4.5g, FRC: 79.5 ± 3.9, GAL: 56.5 ± 3.9), with no difference in protein (GLC: 99.3 ± 2.5g, FRC: 105.5 ± 2.9, GAL: 100.1 ± 3.2) or fat-free dry mass (GLC: 132.1 ± 4.4 g, FRC: 138.7 ± 3.5, GAL: 134.2 ± 4.2) between these three groups. Rats on the GOS diet had significantly higher body (GOS: 520 ± 11.2 g, MC: 481 ± 11.0), fat (GOS: 64.7 ± 6.7 g, MC: 50.9 ± 4.0), protein (GOS: 102.7 ± 2.5 g, MC: 93.6 ± 2.0) and fat-free dry masses (GOS: 137.6 ± 3.2 g, MC: 124.8 ± 3.2) than rats fed MC.

### Hyperinsulinemic-euglycemic clamp

**Fig 1:**
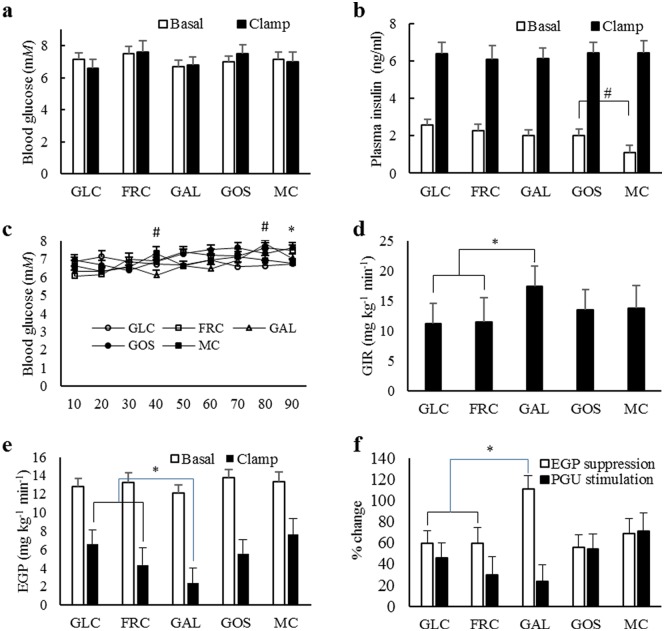
Basal and hyperinsulinemic-euglycemic clamp results from rats fed diets containing 15% glucose (GLC), fructose (FRC), galactose (GAL), galacto-oligosaccharides (GOS) or methylcellulose (MC) for 9 weeks. (A) Blood glucose concentration during basal and clamp periods. (B) Plasma insulin concentrations during basal and clamp periods. (C) Blood glucose concentrations during last 90 minutes of the clamp period. (D) Glucose infusion rate during clamp. (E) EGP during basal and clamp periods. (F) Percent suppression of EGP and stimulation of PGU by insulin. Values are least-square means ± standard error; n = 8. *P < 0.05 for GAL vs GLC and FRC contrast. #*P* < 0.05 for GOS vs. MC contrast.

MC intake lowered basal plasma insulin in comparison to GOS, GLC and FRC, with no difference in basal plasma glucose or glucose flux (Fig 1A and 1B). Steady-state GIR during the clamp period was 53% greater with GAL treatment in comparison to GLC and FRC (Fig 1D), indicating an improvement in whole-body insulin sensitivity. A 57% decline in EGP with GAL over GLC and FRC (Fig 1E), and greater suppression by insulin (Fig 1F), implicates the liver in the whole-body effect. Stimulation of PGU by insulin was not affected by GAL (Fig 1F), which means that all of the whole-body response to GAL can be attributed to an improved hepatic insulin sensitivity. GOS and MC did not differ in any of the clamp parameters, signifying no effect of GOS on insulin sensitivity in either liver or periphery.

Glucose uptake index (Rg) was highest in cardiac tissue and iBAT (Table 1). Soleus, gastrocnemius and diaphragm muscles and brain exhibited similar indices while omental white adipose had the lowest Rg. GAL did not affect Rg in any tissue, supporting the conclusion from [^3^H]glucose kinetics that peripheral insulin sensitivity was not altered. GOS tended to decrease Rg in diaphragm, soleus, gastrocnemius and iBAT, although the iBAT effect disappeared when corrected for UCP1 content of the iBAT sample.

**Table 1:** teady-state tissue glucose uptake index (R_g_; mM mg^-1^ min^-1^) from 2-[1-^14^C] deoxyglucose disappearance.

	Diet						
	GLC	FRC	GAL	GOS	MC	*P*_*GAL v GF*_	*P*_*GOS v MC*_
Diaphragm	7.35±0.76	8.79±1.57	8.75±1.63	7.79±1.19	10.11±2.42	0.37	0.09
Soleus	3.10±0.64	2.98±1.11	2.62±0.43	1.93±0.58	2.74±0.72	0.94	0.06
Gastrocnemius	1.27±0.32	2.61±0.91	2.30±0.48	1.39±0.52	2.51±0.85	0.90	0.07
Om. adipose	29.7±4.34	30.5±5.52	33.8±4.57	27.9±2.77	33.8±6.46	0.67	0.64
Brain	10.4±0.45	9.19±0.93	15.9±6.12	9.37±0.90	12.1±1.25	0.12	0.45
iBAT	16.6±10.9	22.1±10.1	31.6±17.0	16.3±6.3	30.7±9.2	0.15	0.03
iBAT/UCP1	23.9±10.2	38.2±20.3	48.5±19.5	32.1±11.8	42.1±15.9	0.47	0.64
Brain	10.4±0.45	9.19±0.93	15.9±6.12	9.37±0.90	12.1±1.25	0.12	0.45

Abbreviations: FRC, diet with 15% fructose; GAL, diet with 15% galactose; GLC, diet with 15% glucose; GOS, diet with 15% galacto-oligosaccharide; iBAT, interscapular brown adipose tissue; iBAT/UCP1, R_g_ for iBAT adjusted for uncoupling protein 1 detected by western blot; MC, diet with 15% methylcellulose; *P*_GAL v GF_, *P*-value for GAL vs GLC and FRC contrast; *P*_GOS v MC_, *P*-value for GOS vs. MC contrast. Data are means ± SE, n = 7.

### Fecal microbiome

Monosaccharide intake affected fecal abundance of *Clostridium coccoides* and Firmicutes (Table 2). GAL lowered Firmicutes abundance by 17% and the ratio of Firmicutes:Bacteroidetes by 70% in comparison to the combined effects of GLC and FRC.

**Table 2:** Quantification of gut microbial populations (arbitrary units) by RT-qPCR of fecal DNA.

	Diet						
	GLC	FRC	GAL	GOS	MC	*P*_*GAL v GF*_	*P*_*GOS v MC*_
Bacteroidetes	0.14±0.04	0.27±0.10	0.38±0.07	1.93±0.46	1.03±0.09	0.34	<0.01
*Bifidobacterium*	53.8±13.1	59.3±10.1	169±122	1204±137	2.5±0.89	0.20	<0.01
*C*. *coccoides* (Firmicutes)	2.28±0.19	1.51±0.13	1.55±0.17	1.50±0.20	1.02±0.08	0.04	0.08
Enterobacteriaceae	1.16±0.28	0.98±0.14	1.61±0.42	0.38±0.12	1.17±0.23	0.20	0.07
Firmicutes	1.69±0.09	1.37±0.05	1.27±0.06	1.11±0.07	1.01±0.05	<0.01	0.17
*Lactobacillus* (Firmicutes)	0.90±0.17	1.11±0.17	1.17±0.26	2.43±0.82	1.09±0.15	0.61	0.02
Firm:Bdetes	16.2±0.81	9.51±1.52	3.83±0.59	0.66±0.71	1.00±0.53	<0.01	0.64

Abbreviations: Firm:Bdetes, ratio of Firmicutes to Bacteroidetes; FRC, diet with 15% fructose; GAL, diet with 15% galactose; GLC, diet with 15% glucose; GOS, diet with 15% galacto-oligosaccharide; MC, diet with 15% methylcellulose; *P*_GAL v GF_, *P*-value for GAL vs GLC and FRC contrast; *P*_GOS v MC_, *P*-value for GOS vs. MC contrast. Data are means ± SE, n = 7.

The GOS diet increased *Bifidobacterium* spp. 481-fold, Bacteroidetes by 86%, and *Lactobacillus* spp. 1.2-fold compared to intake of the non-digestible, non-fermentable MC. In addition, GOS tended to increase *Clostridium coccoides* by 47% and decrease Enterobacteriaceae by 67% over MC.

### Colon and liver responses

Fed-state proximal colon expression levels of glucose transporter 1 (GLUT1) and Na-dependent glucose transporter 1 (SGLT1) were decreased by GAL consumption, compared to GLC and FRC, while expression of GLUT2 tended to increase (Table 3). GOS intake tended to increase GLUT2 and mucin 3 (MUC3) expression relative to MC.

**Table 3:** Proximal colon gene expression analysis by RT-qPCR (arbitrary units).

	Diet						
	GLC	FRC	GAL	GOS	MC	*P*_*GAL v GF*_	*P*_*GOS v MC*_
GLP-1 precursor and secretion							
Proglucagon	0.11±0.84	0.40±0.78	0.88±0.56	0.77±0.54	0.3±0.84	0.11	0.41
Proprotein convertase 1	1.45±0.20	0.97±0.19	1.31±0.19	1.17±0.15	0.88±0.19	0.64	0.34
Proprotein convertase 2	2.75±0.32	0.85±0.31	1.59±0.31	1.21±0.25	0.71±0.31	0.90	0.26
Enterocyte glucose transporters							
GLUT1	2.41±0.18	2.58±0.18	1.62±0.17	1.39±0.14	1.61±0.19	0.039	0.61
GLUT2	4.51±0.69	3.95±0.66	16.5±0.66	6.9±0.54	0.89±0.92	0.08	0.09
GLUT5	2.51±0.38	1.72±0.36	1.36±0.36	1.53±0.29	1.32±0.50	0.30	0.81
SGLT1	1.94±0.23	1.43±0.21	0.98±0.22	1.07±0.18	1.22±0.30	0.04	0.73
Gut barrier mucins							
Mucin 2	1.85±0.29	1.41±0.29	1.08±0.27	1.19±0.22	1.03±0.22	0.20	0.73
Mucin 3	2.87±0.19^a^	1.32±0.18^ab^	1.91±0.19^ab^	1.55±0.15^ab^	0.90±0.22^b^	0.93	0.07
Mucin 4	0.85±0.33	0.53±0.31	0.96±0.31	0.73±0.25	0.87±0.37	0.32	0.71

Abbreviations: FRC, diet with 15% fructose; GAL, diet with 15% galactose; GLC, diet with 15% glucose; GLUT, glucose transporter; GOS, diet with 15% galacto-oligosaccharide; MC, diet with 15% methylcellulose; *P*_GAL v GF_, *P*-value for GAL vs GLC and FRC contrast; *P*_GOS v MC_, *P*-value for GOS vs. MC contrast; SGLT1, Na-dependent glucose transporter 1. Data are means ± SE, n = 6.

**Fig 2:**
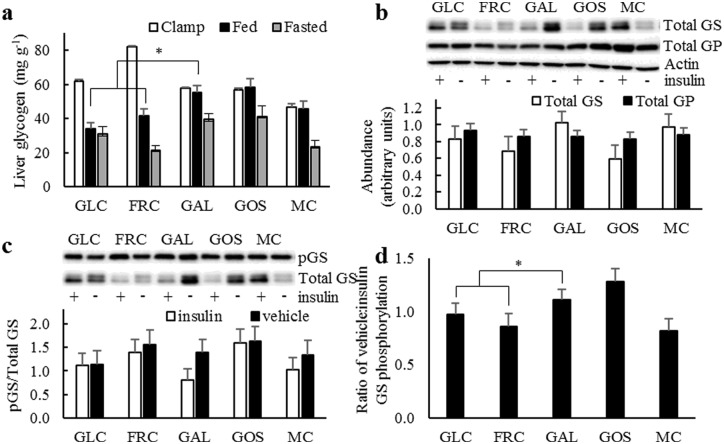
Hepatic glycogen results from rats fed diets containing 15% glucose (GLC), fructose (FRC), galactose (GAL), galacto-oligosaccharides (GOS) or methylcellulose (MC) for 9 weeks. (A) Liver glycogen content (n = 7) following the hyperinsulinemic-euglycemic clamp, after an overnight fast, or 3 h post-feeding. (B) Hepatic glycogen synthase (GS) and glycogen phosphorylase (GP) protein abundances relative to β-actin (n = 12). (C) Phosphorylation state of hepatic GS 8 min after i.p. injection of insulin or vehicle (n = 6). (D) Insulin effect on hepatic GS phosphorylation expressed as a ratio of phosphorylation state in vehicle-stimulated rats to phosphorylation state in insulin-stimulated rats (n = 6). *P < 0.05 for GAL vs GLC and FRC contrast.

Fasted- and clamp-state hepatic glycogen content (Fig 2A) did not differ across dietary treatments. However, 3 hours post-feeding, hepatic glycogen content was 21% greater with GAL compared to GLC and FRC. GOS intake tended to increase fed-state glycogen content by 13% compared to MC.

Hepatic glycogen phosphorylase abundance did not differ across dietary treatments while glycogen synthase tended to decrease with GOS compared to MC (Fig 2B). Vehicle- and insulin-stimulated phosphorylation of glycogen synthase did not differ (Fig 2C), but insulin’s ability to suppress inhibitory phosphorylation of GS was significantly greater with GAL compared to GLC and FRC (Fig 2D).

## Discussion

The objective of this study was to evaluate the effects of milk sugars on insulin sensitivity when given at 15% of dry matter intake. Galactose, a monomer of the milk disaccharide lactose, was compared to fructose and glucose, the monosaccharide moieties of the plant disaccharide sucrose. Glucose catabolism is regulated in insulin-responsive tissues through insulin activation, as well as intracellular ATP and citrate inhibition, of the glycolytic enzyme phosphofructokinase (EC 2.7.1.11), preventing unnecessary, irreversible glucose breakdown. In contrast, dietary fructose is exclusively catabolized in the liver where fructokinase (EC 2.7.1.4) and aldolase (EC 4.1.2.13) split fructose into 2 triose phosphates, bypassing the highly regulated phosphofructokinase step [23] and yielding acetyl-CoA building blocks for *de novo* lipogenesis more readily than glucose. Thus, high fructose intake is a major contributing factor to the obesity epidemic [24] and has been exploited to experimentally create hypertriglyceridemia-induced insulin resistance [25,26].

Galactose is metabolized in the liver where epimerization to glucose occurs while attached to UDP, yielding UDP-glucose. UDP-glucose is the immediate precursor for glycogen synthesis and is not readily reversed through UDP-glucose pyrophosphorylase (EC 2.7.7.9) to glucose-1-phosphate *in vivo*. Therefore, in order for galactose to contribute to blood glucose, it must first enter into glycogen and then be released via glycogenolysis. As a consequence of this pathway, which is perhaps one of the evolutionary advantages of galactose in milk sugars, virtually 100% of dietary galactose absorbed from the gastrointestinal tract is converted to hepatic glycogen [12]. There is an upper limit to the concentration of glycogen that can be maintained in hepatocytes [27] so glycogen synthesis from galactose may reduce incorporation of glucose into glycogen. Adding galactose to a diet can induce hyperglycemia [28] by sparing glucose from entry into glycogen, but isocaloric substitution of galactose for glucose does not affect glycemia [29]. Although inefficient hepatic uptake of galactose from diets containing > 30% galactose leads to hypergalactosemia that is used to mimic symptoms of diabetes [30], whether the preferential diversion of galactose into hepatic glycogen influences the sensitivity of glucose flux to insulin has not been investigated previously.

Here we report that isocaloric substitution of galactose for glucose or fructose, at 15% of dietary dry matter, improved hepatic insulin sensitivity with no alteration in plasma glucose concentration. Similarly, replacement of starch with 50% galactose did not affect plasma glucose [29]. To our knowledge, there has been little evaluation of the effect of galactose on insulin sensitivity. Pantophlet et al. [31] found no difference between glucose, fructose or lactose consumed at 15% of dry matter intake by young cattle in the QUICKI index of hepatic insulin sensitivity estimated from fasting plasma glucose and insulin concentrations. Our study is the first to show, with the hyperinsulinemic-euglycemic clamp, that galactose can increase hepatic insulin sensitivity. We also found increased hepatic glycogen content three-hours post-feeding in response to GAL intake compared to GLC or FRC, which was associated with a GAL-induced enhancement of the effect of insulin on the proportion of glycogen synthase in the non-phosphorylated, active state, and no change in glycogen phosphorylase abundance. Consistent with our results, oral gavage of galactose compared to glucose led to faster hepatic glycogen accumulation [32] and prolonged activatory dephosphorylation of glycogen synthase [33] in rats. Active glycogen synthase is required for hepatic clearance of galactose from blood [12] but it also incorporates glucose-derived carbon into glycogen. It is possible that the more potent activation of hepatic glycogen synthase during GAL treatment accounts for the increased hepatic insulin sensitivity we observed.

Despite activation of glycogen synthase following galactose gavage in the experiment of Niewohner and Niel [33], hepatic glycogen accumulated more rapidly after a glucose dose and it was suggested that the capacity for hepatic uridylation of galactose can be exceeded at high doses of galactose, leading to hypergalactosemia and loss of galactose into urine. Inclusion of galactose into diets at greater than 30% of dry matter content induces hypergalactosemia and symptoms of diabetes. Thus, the improvement in hepatic insulin sensitivity from galactose consumed at 15% of dry matter intake may not be maintained at higher galactose intakes.

To our knowledge, this is the first study showing effects of galactose on the fecal microbiome. There were decreases in fecal *C*. *coccoides* and total Firmicutes counts and the Firmicutes: Bacteroidetes ratio in comparison to GLC and FRC. A high Firmicutes:Bacteroidetes ratio is associated with obesity, and prebiotic fibres decrease the ratio, resulting in higher circulating GLP-1 [34]. The tendency for GAL to increase colonic proglucagon expression may provide a link between intestinal microbiome effects and improved insulin sensitivity although basal plasma insulin was not affected.

In contrast to galactose, galacto-oligosaccharides from milk are indigestible but exert prebiotic effects, which can increase colonic short-chain fatty acid production [35] and ameliorate high-fat diet induced endotoxemia, thereby improving inflammatory status and glucose tolerance [36,37]. In this study, the effects of GOS intake were compared to MC which serves as a negative control since it is also indigestible, but non-fermentable, while GOS is indigestible and fermentable.

Prebiotic GOS decreased Firmicutes while increasing Bacteroidetes, *Bifidobacterium* and *Lactobacillus* spp. in comparison to MC. In particular, the bifidogenic effect is typical of oligosaccharide intake [17], with as little as 1% w/w dietary galacto-oligosaccharides resulting in a 9% increase in *Bifidobacterium* [38]. Bifidogenic effects were observed after just 7 days of consumption of a diet containing 5% inulin versus cellulose [39]. This shift in microbial populations is associated with increased GLP-1 secretion from intestinal L-cells and improved glucose tolerance [40]. Supplementing diets containing 72% of calories from fat with 10% oligofructose improved hepatic insulin sensitivity via increased insulin-stimulated phosphorylation of IRS1 and Akt [41], along with increased intestinal proglucagon expression and no effect on peripheral insulin sensitivity. However, oligofructose supplementation on diets containing 58% of calories from fat did not alter colonic proglucagon expression nor blood glucose or insulin concentrations [42]. An anti-diabetic effect on only the highest of high-fat diets may explain why the inclusion of 15% GOS in our diet, providing just 35% of calories from fat, did not alter insulin sensitivity or colonic proglucagon expression.

The tendencies for increased colonic GLUT2 and mucin 3 expression are evidence of a prebiotic response to GOS at 15% of intake. Mucins, produced by goblet cells, form a protective layer over mucosal surfaces. In the gastro-intestinal tract, mucins 3 and 4 are membrane-bound, while mucin 2 is secreted and forms a gel-like barrier on the apical side of intestinal epithelial cells [43]. Like us, Paturi et al. [39] reported that colonic mucin 3 expression increased with 5% dietary inulin while mucins 2 and 4 were unaffected. However, mice fed 5% galacto-oligosaccharide increased ileal mucin 2, but not mucin 4 expression [44]. These GOS effects on intestinal barrier function may be related to bifidogenesis or direct modulation of intestinal goblet cells by galacto-oligosaccharides [45].

Despite improvements in colonic microbial profile, barrier function and proglucagon expression, GOS had no effect on GIR, EGP or PGU during the clamp. Furthermore, GOS tended to decrease glucose uptake indices in diaphragm, soleus and gastrocnemius muscles. Diets containing 33% fructose instead of glucose elicited a similar decrease in glucose uptake indices [26]. Glucose transport into skeletal muscle and adipose is insulin-responsive so the low 2-DOG uptakes indicate peripheral insulin resistance. To our knowledge, this study is the first assessment of *in vivo* glucose uptake using the 2-DOG tracer in response to oligosaccharide intake.

In conclusion, of the milk sugars, galactose at 15% of daily intake improved hepatic insulin sensitivity in non-diabetic, normal rats compared to glucose and fructose as assessed by the hyperinsulinemic-euglycemic clamp. Galactose caused an increase in fed-state hepatic glycogen content and the ability of insulin to suppress phosphorylation of glycogen synthase and also caused a favourable shift in gut microbial populations. Intake of galacto-oligosaccharides improved the gut microbial profile and colonic gene expression but did not improve insulin sensitivity in the liver or periphery. These results indicate that milk sugars, particularly the galactose moiety of lactose, has beneficial effects on insulin sensitivity while the more commonly consumed glucose and fructose had negatively affected insulin sensitivity. Further research is required to delineate under which circumstances the beneficial prebiotic effects of galacto-oligosaccharides can translate into improved host metabolism, particularly in regards to insulin sensitivity.

## Supporting information

S1 Table Primers used for proximal colon gene expression and fecal DNA analyses. Abbreviations: Bdetes, Bacteroidetes; Bifido, Bifidobacterium; C. cocc, Clostridium coccoides; Enterob, Enterobacteriaceae; Firm, Firmicutes; GLUT, glucose transporter; Lbacil, Lactobacillus; MUC, mucin; PC, proprotein convertase; ProG, proglucagon; SGLT1, Na-dependent glucose transporter 1.
(PDF)

## Abbreviations

2-DOG: 2-[1-^14^C]deoxyglucose
AUC: area-under-the-curve
EGP: endogenous glucose production
Firm:Bdetes: rat of Firmicutes to Bacteroidetes
FRC: fructose
GAL: galactose
GIR: glucose infusion rate
GLC: glucose
GLUT: glucose transporter
GOS: galacto-oligosaccharides
GP: glycogen phosphorylase
GS: glycogen synthase
iBAT: interscapular brown adipose tissue
MC: methylcellulose
MUC3: mucin 3
pGS: phosphorylated glycogen synthase
PGU: peripheral glucose utilization
Rg: glucose uptake index
SGLT1: sodium-dependent glucose transporter 1
UCP1: uncoupling protein 1
