# Supplementary material for: Of the milk sugars, galactose, but not prebiotic galacto-oligosaccharide, improves insulin sensitivity in male Sprague-Dawley rats
Source: PLoS One. 2017 Feb 16;12(2):e0172260. doi: 10.1371/journal.pone.0172260 (PMC5313224; doi:10.1371/journal.pone.0172260)
Supplement: S1 Table — Abbreviations: Bdetes, Bacteroidetes; Bifido, Bifidobacterium; C. cocc, Clostridium coccoides; Enterob, Enterobacteriaceae; Firm, Firmicutes; GLUT, glucose transporter; Lbacil, Lactobacillus; MUC, mucin; PC, proprotein convertase; ProG, proglucagon; SGLT1, Na-dependent glucose transporter 1. (PDF) [file pone.0172260.s001.pdf]

S1. Primers used for proximal colon gene expression and fecal DNA analyses.

Gene	Forward primer 5’ - 3’	Reverse primer 5’ - 3’	NCBI gene ID
Proximal colon			
β-actin	CTGACCGAGCGTGGCTACAG	GGTGCTAGGAGCCAGGGCAG	NM031144.3
GLUT1	CTGTAGGGCTGGACCTTTGG	AATGGAGCCTGGACCCCTAT	NM_138827.1
GLUT2	GTTGCTGGATAAGTTCACCTGGAT	GATTGGACCTGGCCCAATCT	XM_006232207.2
GLUT5	GGCCTCATCTTCCCATTCATTCAA	GGTAGCAGGTGGGAGGTCATTA	D28562.1
MUC2	GCAAGGACTGTGTTTGCCTG	TTCAGCTTTGCACCGTTTGG	XM_008760048.1
MUC3	CTTGAGGAGGTGTGCAAGAAA	CCCCAGGGTGACATACTTTG	XM_008769185.1
MUC4	GCTTGGACATTTGGTGATCC	GCCCGTTGAAGGTGTATTTG	XM_006248471.2
PC1	GGTACCCAAAAACTCCAGCA	GGCTTGTTGAGCTTTTCCAG	XM_008773859.1
PC2	TCGATCAGGTGGTGAGGGAT	GCGTGGCCCTAGTTCTTTCT	NM_012746.1
ProG	ACCGCCCTGAGATTACTTTTCTG	AGTTCTCTTTCCAGGTTCACCAC	NM_012707.2
SGLT1	CTACATCCAGTCCATCACCCAGTTAC	CCAATCAGGAAGCCGAGAATCAG	NM_013033.2
Fecal DNA			

Bdetes	GAAGGTCCCCCACATTG	CAATCGGAGTTCTTCGTG
Bifido	CGCGTCYGGTGTGAAAG	CCCCACATCCAGCATCCA
C. cocc	ACTCCTACGGGAGGCAGC	GCTTCTTAGTCARGTACCG
Enterob	CATTGACGTTACCCGCAGAAGAAGC	CTCTACGAGACTCAAGCTTGC
Firm	ATGTGGTTTAATTCGAAG	AGCTGACGACAACCATGCAC
Lbacil	AGCAGTAGGGAATCTTCCA	CACCGCTACACATGGAG
Universal	AGAGTTTGATCCTGGCTCAG	GGTTACCTTGTTACGACTT

Abbreviations: Bdetes, Bacteroidetes; Bifido, *Bifidobacterium*; *C. cocc*, *Clostridium coccoides*; Enterob, Enterobacteriaceae; Firm, Firmicutes; GLUT, glucose transporter; *Lbacil*, *Lactobacillus*; MUC, mucin; PC, proprotein convertase; ProG, proglucagon; SGLT1, Na-dependent glucose transporter 1.
